# Supplementary material for: A Likelihood Approach for Real-Time Calibration of Stochastic Compartmental Epidemic Models
Source: PLoS Comput Biol. 2017 Jan 17;13(1):e1005257. doi: 10.1371/journal.pcbi.1005257 (PMC5240920; doi:10.1371/journal.pcbi.1005257)
Supplement: S1 File — (TAR.GZ) [file pcbi.1005257.s014.tar.gz › HSPH_Online-SI-Revision/MSS10/n100-mild/n100-mild_NEW_table0.pdf]

1	R0	{1.24125, 3.38982, 0.1}	{1.22544, 3.07157, 0.}	{{1.22544, 3.07157, 0.}, {1.22544, 3.07157, 0.}}	{1.27396, 9.38834, 100.}	inf	1822.	1594.23	{1414.11, 1754.99}	12.8339	0.0611857	newly	3621.	3522.88	{3113., 3945.}	7.17316	0.0321642	Reff	0.993708	0.983839	{0.981326, 0.985999}	0.993081	ar	34 926.	33 552.8	{32 247., 34 725.}	4.13509
2	R0	{1.29554, 4.51878, 0.1}	{1.25593, 3.29127, 0.}	{{1.25474, 3.26445, 0.}, {1.25474, 3.26445, 0.}}	{3.05704, 27.1646, 100.}	inf	2837.	1999.37	{1794.22, 2171.51}	29.5251	0.153592	newly	4247.	3923.42	{3554., 4273.}	8.27408	0.0383087	Reff	0.958635	0.93984	{0.936966, 0.941732}	1.9613	ar	42 828.	37 399.9	{36 035., 38 664.}	12.6742
3	R0	{1.2443, 3.64292, 0.1}	{1.24236, 3.17616, 0.}	{{1.22544, 3.07157, 0.}, {1.25474, 3.26445, 0.}}	{1.11749, 12.8128, 100.}	inf	2191.	1962.45	{1757.42, 2158.09}	11.2402	0.0530117	newly	4114.	4050.41	{3650., 4426.}	6.39596	0.0283576	Reff	0.95617	0.957507	{0.944736, 0.968105}	1.06508	ar	37 087.	36 111.1	{34 098., 37 687.}	3.50851
4	R0	{1.38465, 3.43331, 0.1}	{1.31642, 2.30983, 3.92}	{{1.31246, 2.23442, 4.}, {1.31246, 2.23442, 4.}}	{4.92744, 32.7229, 3820.}	inf	3745.	2420.35	{2060.85, 2719.69}	35.3713	0.193672	newly	6729.	5916.29	{5204., 6530.}	12.2715	0.0582785	Reff	0.908206	0.880854	{0.875527, 0.883555}	3.01322	ar	49 511.	43 369.3	{41 807., 44 416.}	12.4047
5	R0	{1.25409, 4.1068, 0.1}	{1.22661, 3.07929, 0.}	{{1.22544, 3.07157, 0.}, {1.22544, 3.07157, 0.}}	{2.19545, 25.0198, 100.}	inf	2471.	1732.3	{1573.02, 1881.6}	29.8948	0.155321	newly	3936.	3662.28	{3272., 3992.}	7.68191	0.035614	Reff	0.961511	0.949502	{0.946765, 0.951087}	1.32511	ar	38 571.	34 575.5	{33 455., 35 622.}	10.3588
6	R0	{1.28821, 4.2016, 0.1}	{1.25582, 3.2276, 0.}	{{1.24751, 2.5364, 0.}, {1.25474, 3.26445, 0.}}	{2.51454, 23.1815, 100.}	inf	2769.	2035.37	{1769.95, 2311.1}	26.4943	0.13729	newly	4394.	3990.76	{3564., 4399.}	10.0341	0.0469248	Reff	0.936864	0.922506	{0.918843, 0.924866}	1.55521	ar	41 468.	37 906.3	{36 571., 39 506.}	8.58896
7	R0	{1.24369, 2.56007, 0.1}	{1.25553, 2.62268, 0.45}	{{1.24751, 2.5364, 0.}, {1.30094, 3.11158, 3.}}	{0.951782, 4.01715, 520.}	inf	2411.	2516.9	{2194.23, 2956.84}	10.0207	0.0412816	newly	6137.	6136.66	{5506., 6832.}	6.8144	0.0296362	Reff	0.952269	0.95996	{0.951797, 0.987178}	0.851393	ar	38 603.	38 820.8	{37 144., 42 553.}	3.44341
8	R0	{1.232, 3.53942, 0.1}	{1.22632, 3.07736, 0.}	{{1.22544, 3.07157, 0.}, {1.22544, 3.07157, 0.}}	{0.572104, 13.0547, 100.}	inf	1816.	1662.26	{1466.11, 1833.28}	9.48429	0.0444715	newly	3274.	3441.93	{3016., 3792.}	8.87202	0.0371424	Reff	0.934361	0.931935	{0.92909, 0.93431}	0.382145	ar	34 555.	34 605.8	{33 288., 35 771.}	2.26277
9	R0	{1.36262, 3.85631, 0.1}	{1.30289, 3.09864, 3.42}	{{1.30094, 3.04996, 3.}, {1.31023, 3.11158, 5.}}	{4.3834, 19.6476, 3320.}	inf	3694.	2827.9	{2579.43, 3038.26}	23.4462	0.116918	newly	6191.	5569.95	{5103., 5995.}	10.2389	0.0475659	Reff	0.926772	0.897432	{0.893353, 0.902252}	3.16585	ar	48 410.	43 475.3	{42 456., 44 508.}	10.1936
10	R0	{1.28702, 4.67968, 1.}	{1.25273, 3.2615, 0.}	{{1.25474, 3.26445, 0.}, {1.25474, 3.26445, 0.}}	{2.66458, 30.305, 100.}	inf	2625.	1792.5	{1564.18, 2053.31}	31.7143	0.167785	newly	3765.	3508.52	{3054., 3964.}	9.00452	0.041679	Reff	0.941571	0.926904	{0.923905, 0.930839}	1.58061	ar	41 092.	36 815.3	{35 359., 38 008.}	10.4077
11	R0	{1.35752, 3.47129, 0.1}	{1.24751, 2.5364, 0.}	{{1.24751, 2.5364, 0.}, {1.24751, 2.5364, 0.}}	{8.10353, 26.9319, 100.}	inf	3108.	2214.51	{1925.95, 2470.04}	28.7482	0.149341	newly	6127.	5517.24	{4774., 6142.}	10.9208	0.0515786	Reff	1.02022	0.94869	{0.945415, 0.952082}	7.01097	ar	44 669.	37 663.4	{36 259., 39 000.}	15.6833
12	R0	{1.35929, 5.83647, 0.1}	{1.28346, 3.92748, 0.}	{{1.28596, 3.98513, 0.}, {1.28596, 3.98513, 0.}}	{5.57855, 32.708, 100.}	inf	3507.	2273.35	{1955.46, 2445.94}	35.1767	0.189874	newly	4123.	3778.75	{3442., 4016.}	8.43827	0.0390147	Reff	0.975236	0.936653	{0.934983, 0.939683}	3.95624	ar	46 715.	39 899.5	{38 086., 40 900.}	14.5896
13	R0	{1.28019, 3.29908, 0.1}	{1.24751, 2.5364, 0.}	{{1.24751, 2.5364, 0.}, {1.24751, 2.5364, 0.}}	{2.55251, 23.1178, 100.}	inf	2522.	1853.91	{1558.76, 2128.25}	26.4903	0.136527	newly	5146.	4615.3	{3905., 5271.}	11.8267	0.0562901	Reff	0.960053	0.943882	{0.940434, 0.947396}	1.68439	ar	40 941.	36 382.2	{35 049., 37 793.}	11.135
14	R0	{1.36661, 3.48318, 0.1}	{1.36035, 3.22945, 3.}	{{1.36035, 3.22945, 3.}, {1.36035, 3.22945, 3.}}	{0.458173, 7.28436, 2900.}	inf	3348.	3215.81	{2976.74, 3456.79}	5.35937	0.0241879	newly	5669.	5981.92	{5494., 6390.}	6.73875	0.0281039	Reff	0.881791	0.87955	{0.876251, 0.88276}	0.313145	ar	48 672.	47 655.7	{46 669., 48 473.}	2.11113
15	R0	{1.35865, 3.84123, 0.1}	{1.30172, 3.03717, 3.72}	{{1.30094, 3.04996, 3.}, {1.31023, 3.11158, 5.}}	{4.19288, 20.9324, 3636.}	inf	3969.	2951.3	{2610.55, 3275.11}	25.6412	0.130668	newly	6860.	6069.74	{5502., 6609.}	11.5997	0.0547646	Reff	0.953229	0.926507	{0.920538, 0.934679}	2.8283	ar	48 598.	43 124.7	{41 594., 44 498.}	11.2624
16	R0	{1.22879, 3.98329, 0.1}	{1.22023, 3.10308, 0.42}	{{1.21159, 3.07157, 0.}, {1.22544, 3.57392, 1.}}	{0.696285, 22.0976, 464.}	inf	1615.	1325.19	{1041.76, 1561.48}	18.3714	0.0940762	newly	2786.	2899.78	{2435., 3239.}	10.0761	0.0424286	Reff	0.98678	0.983441	{0.972118, 0.990026}	0.536883	ar	34 405.	32 004.1	{30 291., 33 715.}	6.97838
17	R0	{1.34925, 3.64524, 0.1}	{1.24751, 2.5364, 0.}	{{1.24751, 2.5364, 0.}, {1.24751, 2.5364, 0.}}	{7.54026, 30.4187, 100.}	inf	3518.	2272.97	{1985.25, 2561.26}	35.3902	0.191663	newly	6327.	5468.86	{4770., 6105.}	13.7256	0.0657713	Reff	0.979043	0.920752	{0.916976, 0.924234}	5.95383	ar	46 871.	38 363.	{37 231., 39 582.}	18.1519
18	R0	{1.33054, 2.25275, 0.1}	{1.29555, 2.10372, 4.}	{{1.27121, 1.91565, 4.}, {1.31246, 2.23442, 4.}}	{2.62992, 6.61526, 3900.}	inf	3041.	2756.45	{2262.85, 3225.44}	12.0412	0.0581932	newly	8646.	7943.61	{6911., 8860.}	9.67765	0.0457683	Reff	0.969272	0.947415	{0.931201, 0.960168}	2.255	ar	44 336.	41 969.4	{38 826., 44 231.}	5.59065
19	R0	{1.28394, 3.69859, 0.1}	{1.25257, 3.21447, 0.}	{{1.24751, 3.07157, 0.}, {1.25474, 3.26445, 0.}}	{2.44667, 13.2442, 100.}	inf	2442.	2032.45	{1806.41, 2242.02}	16.7782	0.0815468	newly	4445.	4172.95	{3724., 4576.}	7.59033	0.0347866	Reff	0.980378	0.961548	{0.959356, 0.965307}	1.92411	ar	39 558.	37 067.	{35 388., 38 380.}	6.31685
20	R0	{1.2883, 2.26493, 0.1}	{1.28485, 2.03346, 3.93}	{{1.27121, 1.91565, 4.}, {1.31246, 2.23442, 4.}}	{1.77461, 13.9601, 3830.}	inf	2636.	2498.76	{1891.02, 3037.12}	14.0908	0.0645444	newly	7800.	8291.99	{6777., 9714.}	12.744	0.0531841	Reff	1.01925	1.01819	{1.00432, 1.04057}	1.39915	ar	40 768.	40 266.7	{37 425., 43 608.}	5.40883
21	R0	{1.34098, 2.78621, 0.1}	{1.34047, 2.91337, 3.26}	{{1.27121, 1.91565, 3.}, {1.36035, 3.22945, 4.}}	{2.17546, 18.9808, 3160.}	inf	3142.	3444.56	{2155.51, 4060.19}	24.1385	0.103977	newly	7245.	7580.36	{6625., 8277.}	9.03271	0.0387439	Reff	0.972707	0.968037	{0.935045, 0.979796}	1.14165	ar	44 605.	45 784.5	{38 946., 48 710.}	8.22448
22	R0	{1.33783, 5.95911, 0.1}	{1.22573, 3.0735, 0.}	{{1.22544, 3.07157, 0.}, {1.22544, 3.07157, 0.}}	{8.37924, 48.4235, 100.}	inf	3350.	1628.16	{1430.25, 1792.79}	51.3983	0.314872	newly	3826.	3355.39	{2958., 3739.}	12.4477	0.059262	Reff	0.988844	0.927091	{0.924403, 0.929295}	6.24495	ar	45 418.	34 494.9	{33 357., 35 624.}	24.0501
23	R0	{1.33359, 3.3069, 0.1}	{1.47099, 5.35606, 3.07}	{{1.47651, 5.4396, 3.}, {1.47651, 5.4396, 3.}}	{10.303, 61.9663, 2970.}	inf	3385.	5782.07	{5563.96, 6090.89}	70.8143	0.231156	newly	6752.	7547.74	{7207., 7830.}	11.8175	0.0482736	Reff	0.971427	1.03617	{1.03411, 1.04163}	6.66513	ar	46 245.	56 467.4	{55 852., 57 553.}	22.1049
24	R0	{1.30588, 2.56123, 0.1}	{1.27121, 1.91565, 4.}	{{1.27121, 1.91565, 4.}, {1.27121, 1.91565, 4.}}	{2.65464, 25.206, 3900.}	inf	2762.	2014.94	{1586.92, 2348.28}	27.0749	0.141998	newly	6606.	6052.89	{4893., 7005.}	11.7643	0.0563358	Reff	0.932189	0.91694	{0.911969, 0.921668}	1.63588	ar	42 383.	38 699.5	{36 955., 40 400.}	8.69089
25	R0	{1.29813, 4.51849, 0.1}	{1.25647, 3.29313, 0.}	{{1.25474, 3.26445, 0.}, {1.25474, 3.26445, 0.}}	{3.2094, 27.1188, 100.}	inf	3099.	2193.	{1960.36, 2410.76}	29.2351	0.15198	newly	4627.	4423.56	{3993., 4849.}	6.89734	0.0314034	Reff	0.990123	0.969722	{0.965915, 0.972102}	2.06046	ar	43 371.	37 701.5	{36 197., 39 009.}	13.0721
26	R0	{1.33231, 3.99953, 0.1}	{1.24751, 2.5364, 0.}	{{1.24751, 2.5364, 0.}, {1.24751, 2.5364, 0.}}	{6.36466, 36.5824, 100.}	inf	3222.	1951.62	{1755.81, 2148.88}	39.4282	0.219247	newly	5352.	4693.57	{4242., 5142.}	12.6784	0.0599859	Reff	0.96366	0.918174	{0.915463, 0.920606}	4.72009	ar	44 733.	37 270.5	{36 217., 38 300.}	16.6824
27	R0	{1.30555, 4.61445, 0.1}	{1.25408, 3.22612, 0.}	{{1.25474, 3.26445, 0.}, {1.25474, 3.26445, 0.}}	{3.94203, 30.0866, 100.}	inf	3092.	2120.68	{1939.66, 2319.11}	31.4138	0.165719	newly	4581.	4310.59	{3939., 4690.}	7.23925	0.0330645	Reff	0.983993	0.957383	{0.955296, 0.95992}	2.70433	ar	42 624.	37 474.9	{36 108., 38 713.}	12.0804
28	R0	{1.26819, 5.34969, 0.1}	{1.22544, 3.07157, 0.}	{{1.22544, 3.07157, 0.}, {1.22544, 3.07157, 0.}}	{3.37119, 42.5841, 100.}	inf	2467.	1519.07	{1322.31, 1698.46}	38.4244	0.212916	newly	3182.	3278.67	{2828., 3665.}	8.97454	0.0383264	Reff	0.987438	0.965766	{0.963566, 0.9681}	2.19478	ar	38 946.	33 361.7	{31 982., 34 764.}	14.3385
29	R0	{1.19925, 3.00394, 0.1}	{1.22445, 3.03986, 0.09}	{{1.22544, 3.07157, 0.}, {1.22544, 3.07157, 0.}}	{2.13464, 3.1719, 184.}	inf	1551.	1609.12	{1363.52, 1825.03}	9.83928	0.0422074	newly	3362.	3620.5	{3211., 4075.}	10.4545	0.0429681	Reff	0.975746	0.995535	{0.993414, 0.998717}	2.03153	ar	31 727.	33 434.5	{32 086., 34 872.}	6.20355
30	R0	{1.35749, 3.75483, 0.1}	{1.35775, 3.22109, 3.08}	{{1.36035, 3.22945, 3.}, {1.36035, 3.22945, 3.}}	{0.380976, 14.2146, 2980.}	inf	3853.	3288.33	{3022.8, 3540.9}	14.6554	0.0695312	newly	6557.	6510.1	{6011., 7026.}	4.61126	0.0202156	Reff	0.934306	0.942147	{0.939441, 0.947393}	1.09634	ar	48 244.	46 521.4	{45 233., 47 476.}	3.57655
31	R0	{1.27131, 4.2161, 0.1}	{1.2398, 3.16608, 0.}	{{1.22544, 3.07157, 0.}, {1.25474, 3.26445, 0.}}	{2.47894, 24.905, 100.}	inf	2579.	2003.29	{1783.76, 2207.81}	22.3231	0.111291	newly	4228.	4244.14	{3809., 4642.}	6.18354	0.026939	Reff	0.992664	0.975183	{0.963006, 0.9872}	1.76102	ar	38 299.	36 154.7	{34 181., 37 834.}	5.73764
32	R0	{1.31827, 6.18119, 0.1}	{1.23047, 3.1212, 0.09}	{{1.22544, 3.07157, 0.}, {1.25474, 3.26445, 0.}}	{6.66003, 49.5048, 184.}	inf	3314.	1612.94	{1418.78, 1769.86}	51.3295	0.314378	newly	3614.	3237.35	{2868., 3561.}	10.9887	0.0515295	Reff	0.960834	0.917767	{0.912465, 0.934537}	4.48226	ar	45 262.	34 984.5	{33 668., 36 205.}	22.7067
33	R0	{1.20398, 1.35167, 0.1}	{1.27121, 1.91565, 4.}	{{1.27121, 1.91565, 4.}, {1.27121, 1.91565, 4.}}	{5.58428, 41.7244, 3900.}	inf	1581.	2415.43	{2021.22, 2815.03}	52.7784	0.18025	newly	7272.	8185.94	{6934., 9390.}	15.0943	0.0599116	Reff	0.95218	0.994745	{0.989385, 0.999633}	4.47028	ar	33 329.	39 170.5	{37 616., 40 718.}	17.5269
34	R0	{1.32776, 3.146, 0.1}	{1.36035, 3.22945, 3.}	{{1.36035, 3.22945, 3.}, {1.36035, 3.22945, 3.}}	{2.4544, 2.65265, 2900.}	inf	3143.	3310.27	{3019.85, 3585.98}	7.02253	0.029263	newly	6043.	6403.09	{5828., 6831.}	7.18567	0.0298021	Reff	0.90054	0.920367	{0.916576, 0.924076}	2.20172	ar	44 872.	47 078.4	{46 015., 48 121.}	4.91703
35	R0	{1.279, 1.11336, 0.1}	{1.30222, 1.35944, 3.}	{{1.30222, 1.35944, 3.}, {1.30222, 1.35944, 3.}}	{1.81542, 22.1022, 2900.}	inf	1968.	2480.82	{1941.88, 3010.5}	29.0066	0.108021	newly	7436.	8630.21	{6941., 10 257.}	20.2382	0.0796475	Reff	0.863977	0.872984	{0.865926, 0.87989}	1.08601	ar	41 033.	43 181.7	{40 955., 45 059.}	5.67027
36	R0	{1.20029, 1.94717, 0.1}	{1.23544, 2.42162, 0.66}	{{1.19264, 2.01465, 0.}, {1.24751, 2.5364, 3.}}	{3.20898, 24.3661, 716.}	inf	1687.	2133.47	{1649.97, 2497.23}	29.2066	0.109417	newly	5772.	5978.84	{5107., 6716.}	10.5797	0.0454096	Reff	0.983182	1.0064	{0.976725, 1.01803}	2.63348	ar	33 234.	36 057.2	{31 634., 38 392.}	10.6946
37	R0	{1.33216, 3.69685, 0.1}	{1.25818, 2.25706, 1.8}	{{1.24751, 1.91565, 0.}, {1.27121, 2.5364, 4.}}	{5.55352, 38.9463, 1810.}	inf	3070.	1765.32	{1282.79, 2167.88}	42.4977	0.248214	newly	5464.	4652.45	{3852., 5309.}	15.976	0.078053	Reff	0.949151	0.912883	{0.899978, 0.928394}	3.82107	ar	45 235.	37 201.1	{35 368., 38 810.}	17.7603
38	R0	{1.25434, 2.04314, 0.1}	{1.27018, 1.79714, 3.79}	{{1.2663, 1.35135, 3.}, {1.27121, 1.91565, 4.}}	{1.26288, 12.0401, 3690.}	inf	2420.	2040.87	{1343.2, 2568.32}	19.0124	0.102273	newly	7800.	7320.12	{5620., 8769.}	13.6331	0.0649416	Reff	0.972916	0.990013	{0.983612, 0.996625}	1.75722	ar	38 662.	37 821.9	{34 807., 39 871.}	4.29349
39	R0	{1.27466, 3.0741, 0.1}	{1.24751, 2.5364, 0.}	{{1.24751, 2.5364, 0.}, {1.24751, 2.5364, 0.}}	{2.12975, 17.4912, 100.}	inf	2301.	1983.44	{1729.97, 2245.6}	14.0494	0.0677272	newly	4560.	4668.19	{4065., 5280.}	8.4511	0.0360977	Reff	0.912975	0.897493	{0.894076, 0.900593}	1.69583	ar	40 181.	37 959.7	{36 628., 39 155.}	5.52831
40	R0	{1.33045, 2.68154, 0.1}	{1.31246, 2.23442, 4.}	{{1.31246, 2.23442, 4.}, {1.31246, 2.23442, 4.}}	{1.35232, 16.6739, 3900.}	inf	3224.	2598.49	{2299.24, 2889.42}	19.4017	0.0953754	newly	6981.	6523.38	{5824., 7229.}	8.21029	0.0379124	Reff	0.885574	0.881808	{0.877891, 0.885676}	0.461682	ar	45 951.	43 640.7	{42 625., 44 716.}	5.02783
41	R0	{1.37003, 2.83984, 0.1}	{1.37302, 2.80438, 3.01}	{{1.37363, 2.81014, 3.}, {1.37363, 2.81014, 3.}}	{0.302518, 1.24851, 2910.}	inf	4061.	4073.28	{3760.17, 4428.37}	5.3206	0.0231721	newly	8819.	9165.05	{8471., 9838.}	5.87992	0.0246768	Reff	0.953239	0.955147	{0.950108, 0.959749}	0.384837	ar	48 926.	49 152.1	{48 120., 50 134.}	1.46309
42	R0	{1.3003, 2.94885, 0.1}	{1.2663, 1.35135, 3.}	{{1.2663, 1.35135, 3.}, {1.2663, 1.35135, 3.}}	{2.61515, 54.1737, 2900.}	inf	3021.	1342.15	{937.807, 1840.88}	55.5726	0.36821	newly	6589.	5455.36	{3829., 7168.}	21.5845	0.113682	Reff	0.931561	0.928458	{0.922142, 0.933521}	0.455327	ar	44 237.	35 486.2	{32 981., 37 753.}	19.7817
43	R0	{1.33756, 2.92653, 0.1}	{1.29011, 2.07519, 3.97}	{{1.27121, 1.91565, 4.}, {1.31246, 2.23442, 4.}}	{3.6699, 29.5044, 3870.}	inf	3675.	2508.22	{1994.85, 3010.46}	31.946	0.173228	newly	8332.	7631.77	{6496., 8863.}	11.0647	0.0523838	Reff	0.990757	0.970581	{0.955949, 0.987774}	2.15069	ar	47 342.	40 650.3	{37 922., 43 665.}	14.1633
44	R0	{1.35801, 2.92276, 0.1}	{1.41666, 3.24201, 1.74}	{{1.37363, 2.81014, 0.}, {1.47608, 3.83841, 3.}}	{4.31901, 15.3927, 1724.}	inf	4090.	4669.92	{3715.34, 5804.92}	19.4193	0.0739222	newly	8641.	9203.83	{8277., 10 035.}	8.46696	0.0349664	Reff	0.933387	0.965059	{0.942354, 0.993859}	3.39316	ar	47 940.	51 996.5	{48 140., 56 832.}	8.58769
45	R0	{1.31342, 4.02649, 0.1}	{1.24773, 2.55825, 0.}	{{1.24751, 2.5364, 0.}, {1.24751, 2.5364, 0.}}	{5.00146, 36.4646, 100.}	inf	3015.	1891.73	{1607.04, 2169.57}	37.256	0.205362	newly	4999.	4681.61	{3987., 5367.}	9.36887	0.0433084	Reff	0.979207	0.944247	{0.940751, 0.947572}	3.57029	ar	42 995.	36 596.2	{34 866., 37 898.}	14.8826
46	R0	{1.37919, 3.75889, 0.1}	{1.28591, 2.58709, 2.03}	{{1.24751, 1.91565, 0.}, {1.36035, 3.22945, 4.}}	{6.76332, 31.1742, 2018.}	inf	3990.	2733.53	{1999.2, 3571.89}	31.4905	0.175789	newly	7235.	6648.36	{5669., 7609.}	10.8744	0.0512596	Reff	1.00539	0.95334	{0.924771, 0.998282}	5.17692	ar	49 157.	41 304.5	{37 632., 47 640.}	15.9744
47	R0	{1.37458, 3.56117, 0.1}	{1.33906, 2.80874, 3.41}	{{1.31246, 2.23442, 3.}, {1.36035, 3.22945, 4.}}	{2.58379, 21.1287, 3310.}	inf	4168.	3249.11	{2337.46, 3916.41}	22.1028	0.117722	newly	7293.	7070.19	{6300., 7664.}	6.1293	0.0277756	Reff	0.937725	0.92564	{0.912998, 0.936101}	1.30248	ar	48 478.	45 662.4	{41 598., 48 556.}	6.03422
48	R0	{1.33393, 3.78296, 0.1}	{1.25302, 2.6325, 0.25}	{{1.24751, 2.5364, 0.}, {1.24751, 2.5364, 0.}}	{6.24312, 32.1968, 340.}	inf	3354.	2127.21	{1767.77, 2362.38}	38.215	0.212023	newly	5927.	4987.68	{4301., 5706.}	16.3189	0.0794122	Reff	0.973742	0.929951	{0.924036, 0.931248}	4.59792	ar	44 885.	37 799.3	{36 182., 38 918.}	16.4942
49	R0	{1.24918, 2.39046, 0.1}	{1.26634, 1.35699, 3.01}	{{1.2663, 1.35135, 3.}, {1.2663, 1.35135, 3.}}	{1.37405, 43.233, 2910.}	inf	1875.	1064.9	{613.426, 1623.28}	43.2305	0.273715	newly	4978.	4923.58	{2916., 7146.}	25.1507	0.118291	Reff	0.958921	0.982355	{0.975246, 0.987969}	2.44383	ar	35 969.	33 157.7	{29 921., 36 153.}	8.35467
50	R0	{1.28281, 2.3032, 0.1}	{1.27121, 1.91565, 4.}	{{1.27121, 1.91565, 4.}, {1.27121, 1.91565, 4.}}	{0.90398, 16.8267, 3900.}	inf	2262.	1883.92	{1489.82, 2219.04}	17.3958	0.08687	newly	6004.	5892.41	{4826., 6986.}	10.7593	0.0483365	Reff	0.938273	0.934597	{0.930282, 0.939432}	0.44926	ar	40 223.	38 101.8	{36 326., 39 819.}	5.34373
